# Experiences in aligning WHO SMART guidelines to classification and terminology standards

**DOI:** 10.1136/bmjhci-2022-100691

**Published:** 2023-08-10

**Authors:** Filippa Pretty, Tigest Tamrat, Natschja Ratanaprayul, Maria Barreix, Nenad Friedrich Ivan Kostanjsek, Mary-Lyn Gaffield, Jenny Thompson, Bryn Rhodes, Robert Jakob, Garrett Livingston Mehl, Özge Tunçalp

**Affiliations:** 1 Metadata, Information Management & Classifications Unit, Australian Institute of Health and Welfare, Canberra, Australian Capital Territory, Australia; 2 UNDP/UNFPA/UNICEF/World Bank Special Program of Research, Development and Research Training in Human Reproduction (HRP), Department of Sexual and Reproductive Health and Research, World Health Organization, Geneva, Switzerland; 3 Department of Digital Health and Innovations, World Health Organization, Geneva, Switzerland; 4 Department of Data and Analytics, World Health Organization, Geneva, Switzerland; 5 PATH, Seattle, Washington, USA; 6 Alphora, Salt Lake City, Utah, USA

**Keywords:** data systems, decision support systems, delivery of health care, informatics, medical records

## Abstract

**Objectives:**

Digital adaptation kits (DAKs) distill WHO guidelines for digital use by representing them as workflows, data dictionaries and decision support tables. This paper aims to highlight key lessons learnt in coding data elements of the antenatal care (ANC) and family planning DAKs to standardised classifications and terminologies (CATs).

**Methods:**

We encoded data elements within the ANC and family planning DAKs to standardised CATs from the WHO CATs and other freely available CATs.

**Results:**

The coding process demonstrated approaches to refine the data dictionaries and enhance alignment between data elements and CATs.

**Discussion:**

Applying CATs to WHO clinical and public health guidelines can ensure that recommendations are operationalised in a digital system with appropriate consistency and clarity. This requires a multidisciplinary team and careful review to achieve conceptual equivalence between data elements and standardised terminologies.

**Conclusion:**

The systematic translation of guidelines into digital systems provides an opportunity for leveraging CATs; however, this approach needs further exploration into its implementation in country contexts and transition into machine-readable components.

## Introduction

With increased investments into digital systems, the adoption of standardised classifications and terminologies (CATs) is critical for establishing clarity and consistency when encoding, documenting and exchanging information on health-related events.^1 2^ Classifications are defined as ‘an exhaustive set of mutually exclusive categories to aggregate data at a pre-prescribed level of specialisation for a specific purpose and used to categorise concepts for the purposes of systematic recording or analysis.^3 4^ Terminology is a set of designations ‘required directly or indirectly to describe health conditions and healthcare activities’ to enable accurate specification and unambiguous communication across health settings.^1–4^ CATs provide a common language to describe the care and treatment of patients using standardised terms. The use of fit-for-purpose and freely available CATs are important for representing information in a consistent manner, enabling the storage, retrieval and meaningful analysis of health information and exchange of information across facilities.^5^

**Figure 1:**
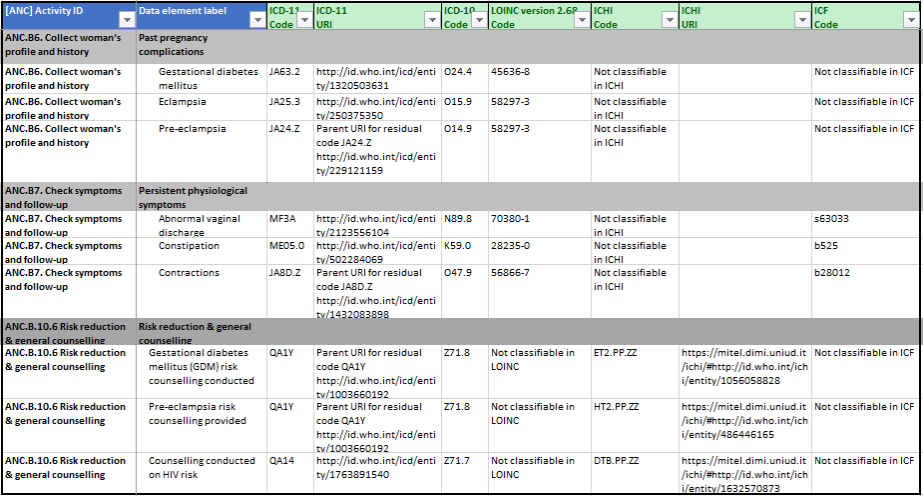
Overview of the ANC DAK data dictionary with data element label, definition and CAT code sets. ANC, antenatal care; CATs, classifications and terminologies; DAK, digital adaptation kit; ICD, International Statistical Classification of Diseases; ICHI, International Classification of Health Interventions; ICF, International Classification of Functioning, Disability and Health; LOINC, Logical Observation Identifier Names and Codes

In 2021, the WHO established the SMART (SMART guidelines stands for standards-based, machine-readable, adaptive, requirements-based and testable) guidelines approach to reinforce clinical, public health and data recommendations through digital systems.^6^ CATs are an integral part of WHO SMART guidelines for ensuring consistency and minimising ambiguity when translating guideline content for digital systems. WHO digital adaptation kits (DAKs), which are one part of the SMART guidelines, define the workflows, core data elements and decision-support logic and other key requirements for digital systems.^6 7^ Each DAK includes a detailed data dictionary containing a comprehensive list of data elements, which are mapped to appropriate, open-access CATs (see figure 1).^8 9^

Despite the value of CATs, the process of incorporating them into point-of-care digital systems may be overlooked or done inadequately for a variety of reasons^1^: perceptions of being resource and time intensive, not understanding the value or return on investment, limited access to clinical terminologists with specialised skillsets and uncertainty in managing mismatches between commonly used medical terms and what is available in established CATs. International public CATs, which are freely available with full functionality, are included in the DAK data dictionaries to overcome challenges associated with the use of these standards. The coded data dictionaries, along with the decision support logic, form the basis for more structured and machine-readable guidance for countries through the use of Health Level Seven Fast Healthcare Interoperability (FHIR) standards.^6^ As such, the data dictionaries within the DAKs are a valuable first step in moving toward the specificity that is needed to support semantic interoperability for meaningful data exchange and continuity of care.

This paper describes the lessons learnt in coding data elements of the antenatal care (ANC)^9^ and family planning DAKs^8^ to standardised CATs and strategies for avoiding common pitfalls and improving the process.

## Methods

WHO teams that developed the DAKs for each health area (ANC or family planning) provided an Excel of the data dictionary with each data element presented in terms of label, data type, input options, validation conditions, skip logics and calculations.^8 9^ Additional columns were included for the classification specialist to provide corresponding code sets for each data element. As the main aim of the SMART guidelines is to accelerate guideline adoption, the DAKs were first drafted based on the clinical health content needs and subsequently aligned to the CATs.

### Coding data elements to CATs

The classification specialist mapped data elements to the CATs listed in table 1 employing online browsers for each code system.^5 10–13^ After an initial coding by the classification specialist, data elements and corresponding concepts were reviewed during virtual meetings with respective health programme experts. Each new DAK was compared against previous DAKs for data element consistency in terms of labels, descriptions and response options. The terminology specialist first reviewed the data dictionaries and proposed a code derived from a search of the CATs based on an understanding of the data element and the description. The health programme leads reviewed the proposed codes through comments on the data dictionary spreadsheet and discussed questions that emerged during weekly calls to resolve issues and ensure the assigned code was as accurate as possible considering the governance and controlled vocabulary constraints of CATs. The final code mappings were approved by the health programme leads.

**Table 1:** Description of the freely available classifications and terminologies used in the DAKs

Classification and terminology type	Publisher	Description
WHO classifications and terminologies		
International Statistical Classification of Diseases and Related Health Problems (ICD-10/ICD-11)	WHO	Defines and classifies diseases, disorders, injuries and other related health conditions, listed in a comprehensive, hierarchical fashion.^5^
International Classification of Health Interventions (ICHI)	WHO	Common tool for reporting and analysing health interventions for statistical purposes.^12^
International Classification of Functioning, Disability and Health (ICF)	WHO	Framework for measuring health and disability at both individual and population levels also includes a list of environmental factors.^13^
Other classifications and terminologies		
Logical Observation Identifier Names and Codes (LOINC)	Regenstrief Institute	Catalogue of measurements, including laboratory tests, clinical measures like vital signs and anthropometric measures, standardised survey instruments and more.^10^
Systematised Nomenclature of Medicine (SNOMED—Global Patient Set)	SNOMED International	Clinical healthcare terminology is designed to provide a core general terminology for electronic health record systems.^11^ SNOMED CT is a propriety code system, and as such the browser used for the coding of the DAKs was the openly available, GPS, which includes a non-hierarchical subset of SNOMED CT codes.

DAKs, digital adaptation kits.

## Results

### Refining consistency and construction of data dictionaries

Coding CATs led to refinements of the data dictionaries to provide greater specificity (eg, ‘diabetes mellitus’ was expanded to include type 1, type 2, gestational and other diabetes mellitus in the ANC DAK data dictionary) or capture a broader range of data. Several data elements, such as ‘nausea/vomiting’, which may be an appropriate data entry from a clinical perspective in pregnancy, were separated into two data elements as each has a different code.

We also reviewed the consistency of data elements within a DAK, and across several DAKs, to standardise the data labels. The effort to reconcile inconsistencies varied based on the amount of data elements in the data dictionary and became incrementally easier as some of the previously reconciled data mappings could be repurposed for new DAK areas.

### Reconciling inexact matches between data elements and code systems

There were frequent instances in which the data element label or description did not match directly with the standardised framing in the CATs. In these cases, we assigned a ‘best fit’ code set for coding purposes, with an explicit indication in the data dictionaries. For example, in the family planning data dictionary, WHO recommendations use the term ‘insulin/non-insulin dependent diabetes’, due to the potential interactions between insulin and hormonal contraception. However, terms available within CATs are ‘type 1/type 2 diabetes’. Ultimately, the insulin/non-insulin dependent categories were retained but coded to type 1/type 2 diabetes as ‘best fit’.

### Usability and applicability of CATs

Each CAT presented unique challenges and depended on clinical term(s) used to search for concepts. Searches generated different responses based on specificity of terms included. For example, if ‘acute’ or ‘chronic’ was added as part of the search, it rendered a different search output compared with when these terms were not included, depending on the granularity of the CATs. As a result, consistency in the search terms and crosschecking different options is necessary to ensure coding to appropriate clinical concepts.

We also observed that some disease conditions and contextual elements were more consistently classifiable using International Statistical Classification of Diseases (ICD-11). In the ANC DAK, International Classification of Functioning, Disability and Health did not have much applicability, which was expected, given that it is a classification for functioning and disabilities, and pregnancy does not traditionally fit in either category. However, some data elements do not need to be coded and will need to be retained simply as the value entered (eg, age, weight) to be useful for clinical purposes. Mapping of demographic (eg, age), chronological (eg, date of visit), contextual data elements (eg, facility location) and descriptions did not align with the structure of CATs. For example, there is no code in the ICD-11 for ‘Gestational Age’ as a general concept. However, there is a standardised range of codes for ‘Duration of Pregnancy’ (https://icd.who.int/dev11/l-m/en%23/http://id.who.int/icd/entity/920837303) which could be applied.

## Discussion

This process uncovered lessons in applying CATs. Achieving conceptual equivalence between the data elements in the data dictionary and reference standards emerged as one of the major learnings and highlighted the need for strengthening linkages between guideline development and health informatics. As clinical and public health guidelines are often not written with health informatics data modelling requirements in mind, the challenges faced are not unexpected. However, this also offers insights on how linkages can be strengthened between these complementary domains to support provision of health services in the digital age while ensuring formal representation through CATs.

The enhanced understanding of the coding relationships to various CATs presents opportunities for refining the process and adhering to the principles and best practice for WHO-FIC Classifications and Terminology Mapping (3). Additionally, this coding exercise would need to be further reviewed when adapting the DAKs to country contexts and into machine-readable guidelines to ensure the consistency in the terms is preserved. Future DAKs could also benefit from a master spreadsheet that standardises data elements repeated across sections, to reduce the time and potential inconsistencies in coding. This would also facilitate the automated creation of FHIR profiles based on the DAK content and codification into machine-readable artefacts in the form of a FHIR Implementation Guide.^14^

## Conclusion

As WHO recommendations are often not conceived with health informaticians, the SMART guidelines approach of systematically applying CATs to WHO clinical and public health recommendations for use in digital systems represents both a daunting and pathfinding effort. Through this endeavour, we highlight mechanisms for leveraging standardised CATs to facilitate meaningful data exchange for continuity of care, measurement, and maximise benefits from countries’ digital implementations.

## Data Availability

No data are available.
